# The current multidisciplinary management of rectal cancer

**DOI:** 10.1002/ags3.12777

**Published:** 2024-01-27

**Authors:** Neal Bhutiani, Oliver Peacock, Abhineet Uppal, Y. Nancy You, Brian K. Bednarski, John M. Skibber, Craig Messick, Michael G. White, George J. Chang, Tsuyoshi Konishi

**Affiliations:** ^1^ Division of Surgery, Department of Colon and Rectal Surgery The University of Texas MD Anderson Cancer Center Houston Texas USA

**Keywords:** lateral pelvic lymph nodes, locally advanced rectal cancer, total neoadjuvant therapy

## Abstract

Multidisciplinary management of rectal cancer has rapidly evolved over the last several years. This review describes recent data surrounding total neoadjuvant therapy, organ preservation, and management of lateral pelvic lymph nodes. It then presents our treatment algorithm for management of rectal cancer at The University of Texas MD Anderson Cancer Center in the context of this and other existing literature. As part of this discussion, the review describes how we tailor management based upon both patient and tumor‐related factors in an effort to optimize patient outcomes.

## Graphical abstract

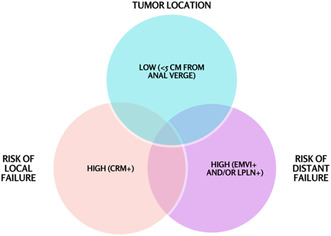

## 1 INTRODUCTION

Over the last 10 years, management of rectal cancer has evolved across multiple fronts. As a result, at The University of Texas MD Anderson Cancer Center, we have experienced a change in our practice across oncologic specialties along these fronts. Applications of multimodal therapy have moved more heavily to the up‐front (preoperative) setting with the advent of total neoadjuvant therapy (TNT). Utilization of the robotic platform for rectal cancer surgery has expanded to include multivisceral resections and locoregional nodal dissection (including lateral pelvic lymph node dissection). Herein, we review each of these developments and how they have influenced our current, multidisciplinary approach to rectal cancer management at MD Anderson Cancer Center.

## 2 TOTAL NEOADJUVANT THERAPY

Regarding the appropriate use of TNT, guidelines from the United States, Europe, and Japan differ. Unlike American guidelines published by the National Comprehensive Cancer Network, which offer TNT as a possible treatment strategy for all T3 or greater or node‐positive rectal cancers, European and Japanese guidelines suggest TNT be used more selectively.^1^ European recommendations suggest TNT for high‐risk, low rectal tumors or those with lateral pelvic lymph node involvement.^2^ Japanese recommendations are based on the principle of upfront surgery with prophylactic lateral pelvic lymph node dissection for tumors below the peritoneal reflection, and reserve chemoradiotherapy only for high‐risk tumors and those that would benefit from pretreatment to improve resectability, without officially mentioning TNT.^3^

The use of TNT at MD Anderson Cancer Center derives from an evaluation of trials that address the risks and benefits associated with TNT, optimal preoperative treatment sequencing, and whether TNT can allow some patients to safely avoid surgery. Among patients with locally advanced rectal cancer, we aim to stratify patients into low‐, intermediate‐, and high‐risk groups based on available data summarized below and patient and tumor‐related factors, employing TNT primarily in intermediate‐ and high‐risk patients. Indeed, we tailor our approach to each patient and their disease to craft a personalized treatment strategy.

## 3 PRODIGE‐23

TNT protocols have most prominently been addressed by two large randomized controlled trials: PRODIGE 23 and RAPIDO.^4^, ^5^ In the PRODIGE 23 trial, which addressed the benefit of treatment intensification, patients with locally advanced rectal cancer receiving TNT (FOLFOXIRI followed by long‐course chemoradiation) demonstrated high treatment compliance and a moderate rate of grade 3–4 adverse events (45%). Compared with patients receiving standard chemoradiotherapy, patients receiving TNT had similar complication rates but less postoperative sexual dysfunction and a higher rate of pathologic complete response (pCR; 27.8% vs. 12.1%). TNT conferred improved 3‐year disease‐free survival (75.7% vs. 68.5%) and a lower rate of distant metastases (17% vs. 25%). The updated long‐term outcomes reported at the 2023 American Society of Clinical Oncology (ASCO) annual meeting showed significant improvement of all outcomes in the TNT arm, with an absolute increase in 5‐year survival of 7.6% for disease‐free survival, 6.9% for overall survival, 9.9% for metastasis‐free survival, and 5.7% for cancer specific survival.^6^ The local recurrence rates were not different between the arms.

The results of this study have led us to incorporate this treatment‐intensified TNT regimen into our practice for patients with locally advanced rectal cancer who have bulky tumors (in an effort to induce maximum regression) and those at high risk of systemic failure (e.g. patients with extramural vascular invasion (EMVI) and those with lateral pelvic lymph node disease). Indeed, we attempt to balance the toxicity of this regimen and lack of data regarding non‐operative management with this approach with the improved disease‐free survival it may confer for these patients, a finding that has been supported by recent work assessing the impact of TNT with induction chemotherapy on disease‐free survival.^7^

## 4 RAPIDO

In the RAPIDO trial, which evaluated whether standard chemotherapy and short course radiation could be used as an effective TNT strategy, patients with locally advanced rectal cancer treated with TNT (short course radiation followed by FOLFOX/CAPOX) exhibited higher rates of pCR (28.4% vs. 14.3%), lower rates of disease‐related treatment failure rate (23.7% vs. 30.4%), and a lower rate of distant metastases (20.0% vs. 26.8%). Again, TNT did not confer a better overall survival benefit compared with standard therapy.

However, recently published 5‐year follow‐up of the RAPIDO trial demonstrated that, at a median follow‐up of 5.6 years, patients treated with TNT exhibited a higher rate of locoregional recurrence (10% vs. 6% standard therapy) that more often breached the mesorectum (21% vs. 4%).^8^ There remained no difference in overall survival between the groups. However, patients treated with TNT continued to demonstrate a similar reduction in disease‐related treatment failure and distant metastases at 5 years. At MD Anderson, while we acknowledge the appeal of short‐course radiation therapy to patients and healthcare systems, the higher rate of locoregional recurrence with the RAPIDO regimen and lack of data regarding its use in non‐operative management has caused us to limit utilization of this short‐course radiation‐based TNT regimen to specific situations (e.g. patients with metastatic disease in whom we aim to provide expeditious local disease control in the pelvis before promptly initiating systemic therapy, patients in whom long‐course chemoradiation is not feasible for logistic reasons).

## 5 STELLAR

The STELLAR trial, a recently‐published trial evaluating a short‐course radiation‐based TNT regimen similar to that used in the RAPIDO trial, compared outcomes among patients with T3‐4 and/or N+ low to mid rectal cancer treated with short‐course radiation followed by four cycles of CAPOX to those treated with conventional long‐course chemoradiation.^9^ Patients treated with TNT exhibited increased rates of pCR (22.5 vs. vs. 12.6% standard therapy). However, local recurrence‐free survival and distant metastasis‐free survival did not differ between the groups. Interestingly, patients treated with TNT experienced improved OS (86.5% vs. 71.5% standard therapy), through rates of acute severe (grade 3–4) toxicity were double in patients treated with TNT (26.5% vs. 12.6% standard therapy). Moreover, the median follow‐up for patients on the trial was less than 3 years (35 months).

The results of this trial, particularly with respect to improved OS despite similar recurrence‐ and distant metastasis‐free survival, are difficult to reconcile with the results from PRODIGE‐23 and RAPIDO detailed above. Moreover, the relatively short follow‐up for patients in the trial limits interpretation of the data. For these reasons, the results of the STELLAR trial have not strongly influenced our treatment algorithms at MD Anderson Cancer Center.

## 6 OPRA

The OPRA trial addressed the question of whether surgery could be safely avoided (i.e. organ preservation, non‐operative management, Watch and Wait) in patients with an excellent clinical response to TNT in light of the pCR rates noted in TNT trials above. Patients with stage II or III rectal cancer were randomized to either FOLFOX chemotherapy followed by long‐course chemoradiation or long‐course chemoradiation followed by FOLFOX. They subsequently underwent either non‐operative management or TME based on tumor response. Preliminary 3‐year disease‐free survival did not significantly differ between the induction (FOLFOX followed by long‐course chemoradiation) and consolidation (long‐course chemoradiation followed by FOLFOX) groups (78% vs. 77%), nor did distant metastasis‐free survival (81% vs. 83%). Consolidation chemotherapy, however, was associated with increased rates of organ preservation at 3 years (58% vs. 43%) with decreased rates of local regrowth once a cCR was achieved (27% vs. 40%).

At MD Anderson Cancer Center, we favor using long‐course chemoradiation followed by consolidation chemotherapy for the majority of our patients with locally advanced rectal cancer, particularly in patients with low rectal cancers who would maximally benefit from non‐operative management. This regimen offers a TNT regimen option that maximizes local tumor treatment response with a standard systemic regimen for patients without high risk for systemic failure. Moreover, we have data specifically addressing the role of this regimen in non‐operative management in patients with rectal cancer as well as its association with a potentially high rate of organ preservation (up to 50%).

A summary of the advantages and disadvantages of the regimens described in the three trials presented above (PRODIGE‐23, RAPIDO, OPRA) can be found in Table 1.

**TABLE 1:** Advantages and disadvantages to total neoadjuvant therapy regimens and current pattern of utilization.

Clinical trial	Regimen description	Advantages	Disadvantages	Target patient at MDACC
PRODIGE‐23	FOLFOXIRI followed by long‐course chemoradiation	Improved disease‐free and overall survival Decreased rates of developing distant metastases	High rate of grade 3–4 adverse events No specific data on use as part of non‐operative management strategy Radiation after chemotherapy allows less time for tumor shrinkage	Aggressive tumors at high risk for systemic failure (i.e. EMVI, multiple lateral pelvic lymph nodes)
RAPIDO	Short‐course radiation followed by FOLFOX	Well‐tolerated Convenience of short‐course radiation	Higher rates of locoregional recurrence compared to standard therapy No specific data on use as part of non‐operative management strategy	Synchronous metastatic disease who require pelvic radiation Unable to receive long‐course chemoradiation
OPRA	Long‐course chemoradiation followed by FOLFOX	Well‐tolerated Radiation prior to chemotherapy allows maximal time for tumor shrinkage Excellent rates of organ preservation	No treatment intensification for patients at high risk of distant metastases	Mid‐ or low‐lying tumors who would like to attempt organ preservation or tumors with positive/threatened CRM, without significant risk for systemic failure in whom triplet chemotherapy is unnecessary.

## 7 MANAGEMENT OF LATERAL PELVIC LYMPH NODES

The management of suspicious lateral pelvic lymph nodes, which we classify as regional disease, in the setting of locally advanced rectal cancer remains an area of active investigation.^10^, ^11^ However, work by our group and others has provided insight that informs our current approach to lateral pelvic lymph nodes.

Work by several groups has helped identify parameters associated with local recurrence in lateral lymph node compartments. Studies from South Korea and the United Kingdom initially identified a higher rate of lateral local recurrence after chemoradiotherapy and total mesorectal excision (TME) among patients with rectal cancer with lateral pelvic lymph nodes ≥10 mm.^12^, ^13^ This was refined by the Lateral Node Study Consortium, which demonstrated that lateral lymph nodes ≥7 mm on initial MRI was associated with a lateral local recurrence rate of nearly 20% after preoperative chemoradiation and TME.^14^ The group also showed that in patients with lateral pelvic lymph nodes ≥7 mm on initial MRI who underwent neoadjuvant chemoradiation, TME, and lateral pelvic lymph node dissection, local recurrence rates in the lateral lymph node basin were 5.7% at 5 years compared to a rate of 19.5% in patients who underwent neoadjuvant chemotherapy and TME alone, findings supported by a subsequent multinational study of patients from the Netherlands, United States, and Australia.^14^, ^15^ This suggests that adding lateral pelvic lymph node dissection to TME after neoadjuvant therapy improves local control in patients with suspicious or enlarged nodes on initial staging.

Identifying selection criteria for patients to undergo lateral pelvic lymph node dissection is further informed by work by our group that assessed radiologic findings of lateral pelvic lymph nodes and pathologic and oncologic outcomes among patients with clinically suspicious lateral pelvic lymph node metastasis who received TNT and underwent lateral pelvic lymph node dissection in addition to TME.^16^ In this cohort of patients with clinical stage II–III rectal cancer with baseline enlarged lateral pelvic lymph node (*n* = 158), lateral pelvic lymph node dissection was performed in 88 patients (56%), and pathological positive lateral pelvic lymph nodes were present in 30 patients (34% of those undergoing lateral pelvic lymph node dissection). The decision to perform lateral pelvic lymph node dissection was made by considering multiple factors, with particular emphasis on baseline malignant characteristics and post‐treatment size ≥5 mm of lateral pelvic lymph nodes, findings that supported previous work from our group assessing patients undergoing LPLND treated only with preoperative chemoradiation.^17^ Finally, retrospective analysis of patients undergoing LPLND at our institution demonstrated that radiographically evident LPLNs on pretreatment MRI rarely occur in patients with primary tumors above the peritoneal reflection and that patients with primary tumors above the peritoneal reflection rarely undergo LPLND.^18^ Although we acknowledge the impact of pre‐treatment size, malignant characteristics, and post‐treatment size, the decision making in clinical practice is often complex and requires incorporation of the primary tumor factors (sidedness, distance from the anal verge, baseline malignant features, response to TNT), time factors (interval from radiotherapy to radiologic assessment), and patient factors (surgical risk).

Finally, multiple studies have demonstrated that minimally invasive lateral pelvic lymph node dissection can be performed with excellent safety and efficacy.^19^, ^20^, ^21^, ^22^ Recent work from our group detailed our experience with 40 patients undergoing robotic lateral pelvic lymph node dissection after preoperative chemoradiation, 90% of whom underwent this dissection with concomitant TME for their primary tumor.^22^ Rate of major morbidity was 10%, a median of six lymph nodes were obtained, and local recurrence rate was 2.5%.

## 8 EVIDENCE‐BASED INDIVIDUALIZED TREATMENT AT MD ANDERSON CANCER CENTER

**FIGURE 1:**
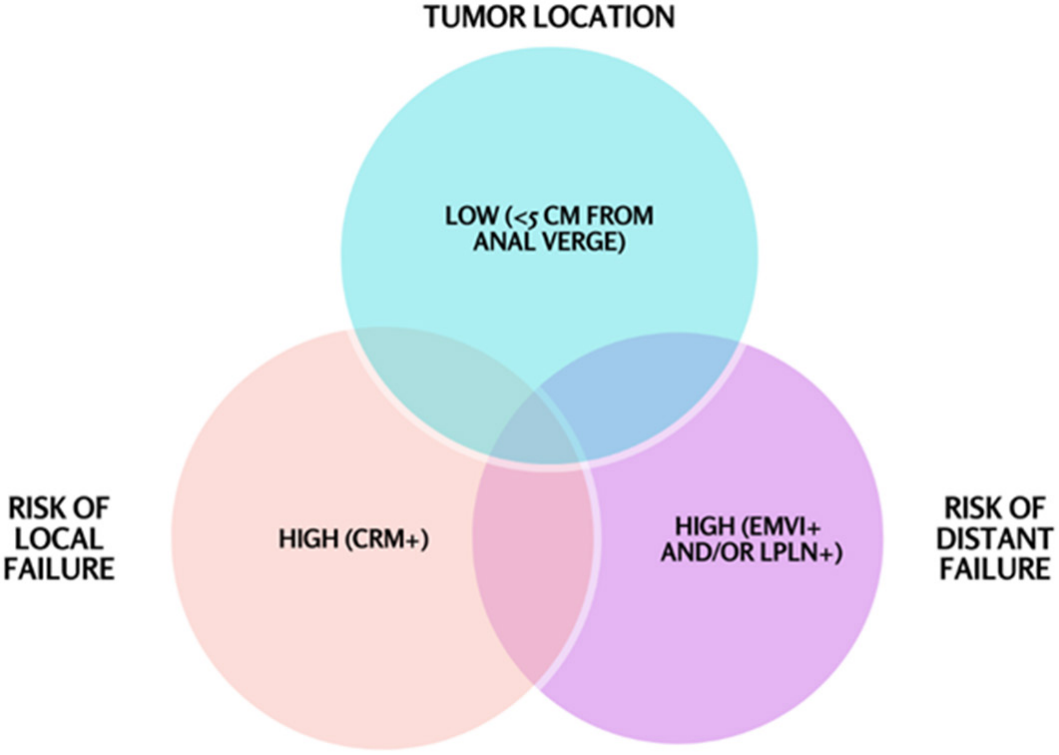
Tumor factors considered in risk‐stratification and decisions regarding total neoadjuvant therapy (TNT). Tumor location, risk of local failure, and risk of distant failure are all considered when deciding about the optimal therapy regimen for a given patient. In general, we strongly consider administering TNT to patients at high risk for local and distant failure, those with low tumors at high risk for distant failure, and those with low tumors at high risk for both local and distant failure.

As previously mentioned, at MD Anderson Cancer Center, we employ TNT selectively, tailoring treatment to tumor‐ and patient‐associated risk factors and treatment goals. Considerations that influence therapy decisions include tumor distance from the anal verge, nodal involvement (including lateral pelvic lymph nodes), circumferential resection margin status, and the presence of EMVI (Figure 1). The MD Anderson approach highlights the importance of high‐quality surgery with TME (and in some cases beyond‐TME approaches as required for margin‐negative resection) and a negative circumferential resection margin (CRM) as well as thoughtful use of chemotherapy and radiation. For all patients, we begin with intensive initial evaluation and staging with high‐quality imaging to allow for appropriate risk stratification (Table 2). This includes serologic evaluation, detailed history and physical examination, and endoscopic evaluation performed by the treating surgeon, along with high‐quality rectal MRI. MRI enables appropriate assessment of the CRM, extramural vascular invasion, distance from the anal verge, and lateral pelvic lymph nodes. Using this information, we stratify patients into low‐risk, intermediate‐risk, and high‐risk groups. In general, in patients who are surgical candidates, we consider margin‐negative resection with adequate lymphadenectomy including a TME and negative CRM as a critical component of rectal cancer treatment. Indeed, the MRC CR07 trial demonstrated a 3‐year local recurrence rate of 4% among rectal cancer patients undergoing TME with negative CRM.^23^ Even among patients with stage III disease who underwent TME with negative CRM, local recurrence rate was only 6%. We use criteria adapted from the QuickSilver, MERCURY, and OCUM trials to identify patients in whom radiation would confer additional benefit from the standpoint of local control.^24^, ^25^, ^26^, ^27^ As an example, the recently published final results from the OCUM trial demonstrated a very low local recurrence rate (4.4% at 5 years) for low‐risk patients (upper rectal tumors, middle and lower rectal tumors without mesorectal fascia involvement, suspicious lymph nodes, or tumor deposits).^27^ This compares favorably with data regarding up‐front surgery in previously published studies (3.3%–5.8%) as well as to similar patients treated with chemoradiation in prospective, randomized trials.^9^, ^28^, ^29^, ^30^ Moreover, based on data from the PROSPECT trial, among patients with low‐risk stage II–III cancer, response to induction FOLFOX informs whether radiation may be safely omitted.^31^ Taken together, we generally omit radiation in patients with clear CRM (≥2 mm), high rectal tumors (those above the peritoneal reflection), and patients with low‐risk mid rectal tumors who demonstrate excellent response to induction chemotherapy and without threatened CRM (Table 3). Similarly, based upon data from several trials assessing the benefit of adjuvant chemotherapy in patients who underwent high‐quality margin‐negative resection, we recommend chemotherapy to patients with node‐positive disease, those in whom we aim to augment response to preoperative chemoradiation, and selectively to those who wish to pursue organ preservation (Table 3).^32^, ^33^, ^34^, ^35^

**TABLE 2:**
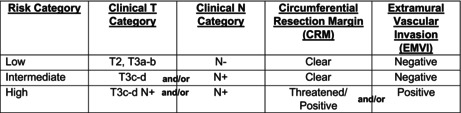
Patient risk‐stratification criteria.

**TABLE 3:** Clinical practice considerations for patients with rectal cancer.

Clinical consideration	Patient population
Avoidance of radiation therapy	Clear CRM (≥2 mm), High rectal tumors (those above the peritoneal reflection) Low‐risk mid rectal tumors who demonstrate excellent response to induction chemotherapy and without threatened CRM
Preoperative chemotherapy	Node‐positive disease Augment response to preoperative chemoradiation Wish to pursue organ preservation (select patients)
Lateral pelvic lymph node dissection	Radiographically abnormal lateral pelvic nodes at the time of diagnosis with nodes ≥5 mm and/or with suspicious malignant features (i.e. irregular borders, internal heterogeneity)

Regarding TNT regimens, among patients with adequate performance status who have aggressive tumors at high risk for systemic failure (i.e. EMVI, multiple lateral pelvic lymph nodes), we favor treatment intensification with triplet induction chemotherapy followed by long‐course chemoradiation as described in the PRODIGE‐23 study due to the survival benefit demonstrated in the trial. For patients with mid‐ or low‐lying tumors or patients with some high‐risk features in whom triplet chemotherapy is deemed unnecessary who would like to attempt organ preservation, we utilize chemoradiation followed by consolidation chemotherapy as described in the OPRA trial. We currently utilize the RAPIDO regimen comprising short‐course radiation therapy followed by CAPOX or FOLFOX only in select patients with synchronous metastatic disease who require pelvic radiation or those unable to receive long‐course chemoradiation (Table 1).

Finally, regarding lateral pelvic lymph nodes, our approach involves assessment of T category and lateral pelvic lymph node appearance on MRI to stratify patients with respect to risk of lateral pelvic lymph node disease.^10^ This stratification then helps determine our approach to lateral pelvic lymph node dissection in the context of tailored therapy. Patients with radiographically abnormal lateral pelvic nodes at the time of diagnosis are restaged after neoadjuvant therapy, and those with nodes ≥5 mm and/or with suspicious malignant features (i.e. irregular borders, internal heterogeneity) undergo lateral pelvic lymph node dissection (Table 3). Patients with lateral pelvic nodes <5 mm without suspicious features after neoadjuvant therapy are, meanwhile, possibly offered observation of their lateral nodes and dissection is deferred.^10^

In summary, treatment of locally advanced rectal cancer at MD Anderson Cancer Center involves a tailored approach that carefully assesses patient and tumor factors. Our approach considers existing data regarding the benefits of systemic chemotherapy, external beam radiation, and TNT with respect to local recurrence, survival, and tumor downstaging and weighs this agains the risks of chemotherapy and radiation. Additionally, we employ TNT in select rectal cancer patients deemed appropriate candidates in which to attempt organ preservation and who are agreeable to attempting this approach. In doing so, our multidisciplinary team aims to optimize patient outcomes from both an oncologic and quality of life perspective.

## FUNDING INFORMATION

No funding was received for this article.

## CONFLICT OF INTEREST STATEMENT

Dr. George J. Chang serves as a member of the editorial board for *Annals of Gastroenterological Surgery*.

## ETHICS STATEMENTS

Approval of the research protocol: N/A.

Informed Consent: N/A.

Registry and the Registration No. of the study: N/A.

Animal Studies: N/A.

## References

[1] Benson AB , Venook AP , Al‐Hawary MM , Arain MA , Chen YJ , Ciombor KK , et al. NCCN guidelines insights: rectal cancer, version 6.2020. J Natl Compr Cancer Netw. 2020;18(7):806–815. 10.6004/jnccn.2020.0032 32634771

[2] Glynne‐Jones R , Wyrwicz L , Tiret E , Brown G , Rödel C , Cervantes A , et al. Rectal cancer: ESMO clinical practice guidelines for diagnosis, treatment and follow‐up. Ann Oncol. 2017;28:iv22–iv40. 28881920 10.1093/annonc/mdx224

[3] Hashiguchi Y , Muro K , Saito Y , Ito Y , Ajioka Y , Hamaguchi T , et al. Japanese Society for Cancer of the Colon and Rectum (JSCCR) guidelines 2019 for the treatment of colorectal cancer. Int J Clin Oncol. 2020;25(1):1–42. 31203527 10.1007/s10147-019-01485-z PMC6946738

[4] Conroy T , Bosset J‐F , Etienne P‐L , Rio E , François É , Mesgouez‐Nebout N , et al. Neoadjuvant chemotherapy with FOLFIRINOX and preoperative chemoradiotherapy for patients with locally advanced rectal cancer (UNICANCER‐PRODIGE 23): a multicentre, randomised, open‐label, phase 3 trial. Lancet Oncol. 2021;22(5):702–715. 33862000 10.1016/S1470-2045(21)00079-6

[5] Bahadoer RR , Dijkstra EA , van Etten B , Marijnen CAM , Putter H , Kranenbarg EMK , et al. Short‐course radiotherapy followed by chemotherapy before total mesorectal excision (TME) versus preoperative chemoradiotherapy, TME, and optional adjuvant chemotherapy in locally advanced rectal cancer (RAPIDO): a randomised, open‐label, phase 3 trial. Lancet Oncol. 2021;22(1):29–42. 33301740 10.1016/S1470-2045(20)30555-6

[6] Etienne P‐L , Rio E , Evesque L , Evesque L , Mesgouez‐Nebout N , Vendrely V , et al. Total neoadjuvant therapy with mFOLFIRINOX versus preoperative chemoradiation in patients with locally advanced rectal cancer: 7‐year results of PRODIGE 23 phase III trial, a UNICANCER GI trial. J Clin Oncol. 2023;41(17_suppl):LBA3504. 10.1016/j.annonc.2024.06.019 38986769

[7] Yamaguchi T , Akiyoshi T , Fukunaga Y , Sakamoto T , Mukai T , Hiyoshi Y , et al. Adding induction chemotherapy before Chemoradiotherapy with total mesorectal excision and selective lateral lymph node dissection for patients with poor‐risk, locally advanced, mid‐to‐low rectal cancer may improve oncologic outcomes: a propensity score‐matched analysis. Ann Surg Oncol. 2023;30:4716–4724. 37032405 10.1245/s10434-023-13458-8

[8] Dijkstra EA , Nilsson PJ , Hospers GAP , Bahadoer RR , Meershoek‐Klein Kranenbarg E , Roodvoets AGH , et al. Locoregional failure during and after short‐course radiotherapy followed by chemotherapy and surgery compared to long‐course chemoradiotherapy and surgery–a five‐year follow‐up of the RAPIDO trial. Ann Surg. 2023;278:e766–e772. 36661037 10.1097/SLA.0000000000005799 PMC10481913

[9] Jin J , Tang Y , Hu C , Cai Y , Zhu Y , Cheng G , et al. Multicenter, randomized phase III trial of short‐term radiotherapy plus chemotherapy versus long‐term chemoradiotherapy in locally advanced rectal cancer (STELLAR). J Clin Oncol. 2022;40(15):1681–1692. 35263150 10.1200/JCO.21.01667 PMC9113208

[10] Peacock O , Chang GJ . The landmark series: management of lateral lymph nodes in locally advanced rectal cancer. Ann Surg Oncol. 2020;27(8):2723–2731. 32519144 10.1245/s10434-020-08639-8

[11] Akiyoshi T , Watanabe T , Miyata S , Kotake K , Muto T , Sugihara K . Results of a Japanese nationwide multi‐institutional study on lateral pelvic lymph node metastasis in low rectal cancer: is it regional or distant disease? Ann Surg. 2012;255(6):1129–1134. 22549752 10.1097/SLA.0b013e3182565d9d

[12] Kusters M , Slater A , Muirhead R , Hompes R , Guy RJ , Jones OM , et al. What to do with lateral nodal disease in low locally advanced rectal cancer? A call for further reflection and research. Dis Colon Rectum. 2017;60(6):577–585. 28481851 10.1097/DCR.0000000000000834

[13] Kim TH , Jeong S‐Y , Choi DH , Kim DY , Jung KH , Moon SH , et al. Lateral lymph node metastasis is a major cause of locoregional recurrence in rectal cancer treated with preoperative chemoradiotherapy and curative resection. Ann Surg Oncol. 2008;15(3):729–737. 18057989 10.1245/s10434-007-9696-x

[14] Ogura A , Konishi T , Beets GL , Cunningham C , Garcia‐Aguilar J , Iversen H , et al. Lateral nodal features on restaging magnetic resonance imaging associated with lateral local recurrence in low rectal cancer after Neoadjuvant Chemoradiotherapy or radiotherapy. JAMA Surg. 2019;154(9):e192172. 31268504 10.1001/jamasurg.2019.2172 PMC6613303

[15] Kroon HM , Malakorn S , Dudi‐Venkata NN , Bedrikovetski S , Liu J , Kenyon‐Smith T , et al. Local recurrences in western low rectal cancer patients treated with or without lateral lymph node dissection after neoadjuvant (chemo)radiotherapy: an international multi‐centre comparative study. Eur J Surg Oncol. 2021;47(9):2441–2449. 34120810 10.1016/j.ejso.2021.06.004

[16] Peacock O , Manisundaram N , Dibrito SR , Kim Y , Hu CY , Bednarski BK , et al. Magnetic resonance imaging directed surgical decision making for lateral pelvic lymph node dissection in rectal cancer after total neoadjuvant therapy (TNT). Ann Surg. 2022;276(4):654–664. 35837891 10.1097/SLA.0000000000005589 PMC9463102

[17] Malakorn S , Yang Y , Bednarski BK , Kaur H , You YN , Holliday EB , et al. Who should get lateral pelvic lymph node dissection after neoadjuvant chemoradiation? Dis Colon Rectum. 2019;62(10):1158–1166. 31490825 10.1097/DCR.0000000000001465

[18] Peacock O , Manisundaram N , Kim Y , Konishi T , Stanietzky N , Vikram R , et al. Therapeutic lateral pelvic lymph node dissection in rectal cancer: when to dissect? Size is not everything. Br J Surg. 2023;110(8):985–986. 37150892 10.1093/bjs/znad115 PMC10361674

[19] Ogura A , Akiyoshi T , Nagasaki T , Konishi T , Fujimoto Y , Nagayama S , et al. Feasibility of laparoscopic total mesorectal excision with extended lateral pelvic lymph node dissection for advanced lower rectal cancer after preoperative chemoradiotherapy. World J Surg. 2017;41(3):868–875. 27730352 10.1007/s00268-016-3762-0

[20] Yamaguchi T , Konishi T , Kinugasa Y , Yamamoto S , Akiyoshi T , Okamura R , et al. Laparoscopic versus open lateral lymph node dissection for locally advanced low rectal cancer: a subgroup analysis of a large multicenter cohort study in Japan. Dis Colon Rectum. 2017;60(9):954–964. 28796734 10.1097/DCR.0000000000000843

[21] Konishi T , Kuroyanagi H , Oya M , Ueno M , Fujimoto Y , Akiyoshi T , et al. Lateral lymph node dissection with preoperative chemoradiation for locally advanced lower rectal cancer through a laparoscopic approach. Surg Endosc. 2011;25(7):2358–2359. 21298544 10.1007/s00464-010-1531-y

[22] Peacock O , Limvorapitak T , Bednarski BK , Kaur H , Taggart MW , Dasari A , et al. Robotic lateral pelvic lymph node dissection after chemoradiation for rectal cancer: a Western perspective. Color Dis. 2020;22(12):2049–2056. 10.1111/codi.15350 32892473

[23] Sebag‐Montefiore D , Stephens RJ , Steele R , Monson J , Grieve R , Khanna S , et al. Preoperative radiotherapy versus selective postoperative chemoradiotherapy in patients with rectal cancer (MRC CR07 and NCIC‐CTG C016): a multicentre, randomised trial. Lancet. 2009;373(9666):811–820. 19269519 10.1016/S0140-6736(09)60484-0 PMC2668947

[24] Kennedy ED , Simunovic M , Jhaveri K , Kirsch R , Brierley J , Drolet S , et al. Safety and feasibility of using magnetic resonance imaging criteria to identify patients with “good prognosis” rectal cancer eligible for primary surgery: the phase 2 nonrandomized QuickSilver clinical trial. JAMA Oncol. 2019;5(7):961–966. 30973610 10.1001/jamaoncol.2019.0186 PMC6583831

[25] Taylor FGM , Quirke P , Heald RJ , Moran BJ , Blomqvist L , Swift IR , et al. Preoperative magnetic resonance imaging assessment of circumferential resection margin predicts disease‐free survival and local recurrence: 5‐year follow‐up results of the MERCURY study. J Clin Oncol. 2014;32(1):34–43. 24276776 10.1200/JCO.2012.45.3258

[26] Ruppert R , Junginger T , Ptok H , Strassburg J , Maurer CA , Brosi P , et al. Oncological outcome after MRI‐based selection for neoadjuvant chemoradiotherapy in the OCUM rectal cancer trial. Br J Surg. 2018;105(11):1519–1529. 29744860 10.1002/bjs.10879

[27] Ruppert R , Junginger T , Kube R , Strassburg J , Lewin A , Baral J , et al. Risk‐adapted neoadjuvant chemoradiotherapy in rectal cancer: final report of the OCUM study. J Clin Oncol. 2023;41(24):4025–4034. 37335957 10.1200/JCO.22.02166

[28] Taylor FG , Quirke P , Heald RJ , Moran B , Blomqvist L , Swift I , et al. Preoperative high‐resolution magnetic resonance imaging can identify good prognosis stage I, II, and III rectal cancer best managed by surgery alone: a prospective, multicenter. Ann Surg. 2011;253(4):711–719. 21475011 10.1097/SLA.0b013e31820b8d52

[29] Lord AC , Corr A , Chandramohan A , Hodges N , Pring E , Airo‐Farulla C , et al. Assessment of the 2020 NICE criteria for preoperative radiotherapy in patients with rectal cancer treated by surgery alone in comparison with proven MRI prognostic factors: a retrospective cohort study. Lancet Oncol. 2022;23(6):793–801. 35512720 10.1016/S1470-2045(22)00214-5

[30] Fleshman J , Branda ME , Sargent DJ , Boller AM , George VV , Abbas MA , et al. Disease‐free survival and local recurrence for laparoscopic resection compared with open resection of stage II to III rectal cancer: follow‐up results of the ACOSOG Z6051 randomized controlled Trial. Ann Surg. 2019;269(4):589–595. 30080730 10.1097/SLA.0000000000003002 PMC6360134

[31] Schrag D , Shi Q , Weiser MR , Gollub MJ , Saltz LB , Musher BL , et al. Preoperative treatment of locally advanced rectal cancer. N Engl J Med. 2023;389:322–334. 37272534 10.1056/NEJMoa2303269 PMC10775881

[32] Hong YS , Kim SY , Lee JS , Nam BH , Kim KP , Kim JE , et al. Oxaliplatin‐based adjuvant chemotherapy for rectal cancer after preoperative chemoradiotherapy (ADORE): long‐term results of a randomized controlled trial. J Clin Oncol. 2019;37(33):3111–3123. 31593484 10.1200/JCO.19.00016

[33] Breugom AJ , van Gijn W , Muller EW , Berglund Å , van den Broek CBM , Fokstuen T , et al. Adjuvant chemotherapy for rectal cancer patients treated with preoperative (chemo)radiotherapy and total mesorectal excision: a Dutch colorectal cancer group (DCCG) randomized phase III trial. Ann Oncol. 2015;26(4):696–701. 25480874 10.1093/annonc/mdu560

[34] Glynne‐Jones R , Counsell N , Quirke P , Mortensen N , Maraveyas A , Meadows HM , et al. Chronicle: results of a randomised phase III trial in locally advanced rectal cancer after neoadjuvant chemoradiation randomising postoperative adjuvant capecitabine plus oxaliplatin (XELOX) versus control. Ann Oncol. 2014;25(7):1356–1362. 24718885 10.1093/annonc/mdu147

[35] Sainato A , Cernusco Luna Nunzia V , Valentini V , de Paoli A , Maurizi ER , Lupattelli M , et al. No benefit of adjuvant fluorouracil leucovorin chemotherapy after neoadjuvant chemoradiotherapy in locally advanced cancer of the rectum (LARC): long term results of a randomized trial (I‐CNR‐RT). Radiother Oncol. 2014;113(2):223–229. 25454175 10.1016/j.radonc.2014.10.006

